# A multicenter evaluation of antibacterial use in hospitalized patients through the SARS-Cov-2 pandemic waves

**DOI:** 10.1186/s12879-023-08042-0

**Published:** 2023-02-24

**Authors:** Laura A. Puzniak, Karri A. Bauer, Kalvin C. Yu, Janet A. Watts, ChinEn Ai, Vikas Gupta

**Affiliations:** 1 Merck & Co., Inc., Kenilworth, NJ USA; 2 Becton, Dickinson and Company, 1 Becton Drive, Franklin Lakes, NJ 07417 USA

**Keywords:** Antibiotics, Antimicrobial therapy, Inpatient, Antimicrobial stewardship, COVID-19

## Abstract

**Background:**

Excessive use of antibiotics has been reported during the SARS-CoV-2 pandemic. We evaluated trends in antibiotic use and culture positive Gram-negative (GN)/Gram-positive (GP) pathogens in US hospitalized patients before and during the SARS-CoV-2 pandemic.

**Methods:**

This multicenter, retrospective study included patients from 271 US facilities with > 1-day inpatient admission with discharge or death between July 1, 2019, and October 30, 2021, in the BD Insights Research Database. We evaluated microbiological testing data, antibacterial use, defined as antibacterial use ≥ 24 h in admitted patients, and duration of antibacterial therapy.

**Results:**

Of 5,518,744 patients included in the analysis, 3,729,295 (67.6%) patients were hospitalized during the pandemic with 2,087,774 (56.0%) tested for SARS-CoV-2 and 189,115 (9.1%) testing positive for SARS-CoV-2. During the pre-pandemic period, 36.2% were prescribed antibacterial therapy and 9.3% tested positive for select GN/GP pathogens. During the SARS-CoV-2 pandemic, antibacterial therapy (57.8%) and positive GN/GP culture (11.9%) were highest in SARS-CoV-2-positive patients followed by SARS-CoV-2-negative patients (antibacterial therapy, 40.1%; GN/GP, pathogens 11.0%), and SARS-CoV-2 not tested (antibacterial therapy 30.4%; GN/GP pathogens 7.2%). Multivariate results showed significant decreases in antibacterial therapy and positive GN/GP cultures for both SARS-CoV-2-positive and negative patients during the pandemic, but no significant overall changes from the pre-pandemic period to the pandemic period.

**Conclusions:**

There was a decline in both antibacterial use and positive GN/GP pathogens in patients testing positive for SARS-CoV-2. However, overall antibiotic use was similar prior to and during the pandemic. These data may inform future efforts to optimize antimicrobial stewardship and prescribing.

**Supplementary Information:**

The online version contains supplementary material available at 10.1186/s12879-023-08042-0.

## Background

The coronavirus disease 2019 (COVID-19) pandemic has had a profound impact on US and global healthcare systems and their efforts to manage bacterial infection and limit antimicrobial resistance. While early reports indicate that approximately 68 to 85% of patients hospitalized with COVID-19 receive antibiotic therapy, only 3.5 to 8.1% of hospitalized patients with COVID-19 show evidence of community-acquired bacterial coinfections and an additional 3 to 14% may develop bacterial infections in the hospital [1–4]. The widespread use of antibiotics in the absence of documented bacterial infection has significant implications for the development of antibiotic resistance, the incidence of *Clostridium difficile* infections, and potential adverse events and related toxicities [5–8].

However, most published data on antimicrobial use and the prevalence of bacterial coinfections in patients with COVID-19 were collected early in the pandemic, when relatively little was known about effective management of COVID-19 or the frequency of coinfections in patients with COVID-19 [3, 9]. These studies also preceded the emergence of clinically relevant COVID-19 variants, including the Delta variant, and do not consider how antibiotic prescribing changed over time. Understanding recent patterns in bacterial coinfections and antibiotic prescribing among inpatients can help identify opportunities to improve patient management and antibiotic stewardship efforts.

In this study, we used data from a large database of US hospitals to evaluate trends in antibacterial use and culture-positive Gram-negative and Gram-positive (GN/GP) pathogens in patients hospitalized before the pandemic (between July 2019 and February 2020) and during various waves within the pandemic (between March 2020 and October 2021).

## Methods

### Study design

We conducted a multicenter, retrospective cohort study evaluating data from 271 US facilities included in the BD Insights Research Database (Becton, Dickinson and Company, Franklin Lakes, NJ), a database that includes small and large hospitals in both rural and urban areas throughout the United States (Additional file 1: Table S1). Eligible admissions included hospitalized adult (≥ 18 years) patients with > 1-day inpatient admission and a record of discharge or death between July 1, 2019, and October 30, 2021. The study dataset, which has been previously described [2, 10–13], was approved as a limited, de-identified dataset available for retrospective analysis and was exempted from patient consent by the New England Institutional Review Board in Wellesley, Massachusetts (No. 120180023) and conducted in compliance with Health Insurance Portability and Accountability Act requirements.

All microbiology results were derived from local testing performed by individual microbiology labs within the cohort of hospitals in the database. SARS-CoV-2 infection was defined as a positive polymerase chain reaction (PCR) or antigen test ≤ 7 days prior to hospitalization or up to 14 days after hospitalization. In this study, a pathogen was defined as a microorganism with the potential to cause disease; the association between the microorganism and a clinically relevant infection was not evaluated.

Gram-negative (GN) or Gram-positive (GP) bacteria were identified from blood, the upper and lower respiratory tract, intra-abdominal tissues, skin, wounds, urine, and other sources based on standards of the local laboratories. GN pathogens evaluated included Enterobacterales (*Citrobacter freundii, Escherichia coli, Enterobacter cloacae, Klebsiella aerogenes, Klebsiella pneumoniae, Klebsiella oxytoca, Proteus mirabilis, Providencia stuartii, Serratia marcescens, Morganella morganii*), *Acinetobacter baumannii, Pseudomonas aeruginosa,* and *Stenotrophomonas maltophilia*, while GP pathogens evaluated included *Enterococcus* species, *Staphylococcus aureus*, and *Streptococcus pneumoniae*.

Results likely to be associated with environmental or surveillance cultures (e.g., rectal or nasal swabs) were excluded using a previously described algorithmic methodology that considers the source of the specimen, the time of collection, the type of microorganism, and the number of microorganisms in a culture to help exclude non-pathogenic samples [14].

### Outcomes

The primary outcomes of our study were the percentage of patients who were prescribed an antimicrobial agent for at least 24 h and the percentage of patients with a GN/GP-positive culture result not due to a contaminant. Secondary outcomes included the type of antimicrobial prescribed and the duration of antibiotic usage. Patients were classified into one of four groups:

1. those discharged in the pre-pandemic period (July 2019 to February 2020), and the following 3 groups during the pandemic periods (March 2020 to May 2020 [original virus], June 2020 to August 2020, September 2020 to November 2020, December 2020 to February 2021 [predominantly Alpha variant], March 2021 to May 2021, June 2021 to October 2021 [predominantly Delta variant]);
2. those who tested positive for severe acute respiratory syndrome coronavirus 2 (SARS-CoV-2) infection;
3. those who tested negative for SARS-CoV-2; and
4. those not tested for SARS-CoV-2 (also referred to as “untested” patients).

Antibiotic use was also analyzed by commonly used antibacterial classes (3rd and 4th generation cephalosporins, beta-lactam with beta-lactamase inhibitor combination [BLIC], glycopeptides, and macrolides).

### Statistical analysis

Descriptive analyses were used to evaluate trends in antibiotic use, culture-positive results, and pathogen types. Generalized estimating equations (GEE) were used to assess the percentage of patients using antibiotic therapy and with GN/GP-positive pathogens across the study period and during the pandemic by SARS-CoV-2 testing status and to estimate prevalence with 95% confidence intervals. Trends were evaluated by pandemic wave. All GEE models were adjusted by gender, age, length of stay (LOS), ICU stay, use of ventilators, and ≥ 1 comorbid condition as well as by hospital-level factors (urban vs rural status, bed size, teaching status, and geographic region). In bivariate analyses, chi-square tests were used to evaluate the bivariate correlations of SARS-CoV-2 status with the percentages of patients prescribed antibiotics and percentages of patients with GN/GP pathogens. To evaluate the bivariate relationships between LOS (or GN/GP LOS) and SARS-CoV-2 status, T-tests or analyses of variance (ANOVA) were used. All statistical tests were performed using a pre-specified two-tailed alpha level of 0.05. Analyses were conducted using R (R Ver. 4.1.2, R Foundation for Statistical Computing, Vienna, Austria), with RStudio (Boston, MA).

## Results

There were 1,789,449 patients hospitalized in the 8-month pre-pandemic period (July 1, 2019, to February 29, 2020) and 3,729,295 patients hospitalized during the pandemic period (March 1, 2020, to October 30, 2021) (Table 1). During the pandemic period, 2,087,774 were tested for SARS-CoV-2 (56.0%) and 189,115 (9.1%) tested positive for SARS-CoV-2 (Table 2). Mean (standard deviation [SD]) age was significantly higher in the SARS-CoV-2-positive group (61.5 [17.9] vs 58.5 [19.7] years for SARS-CoV-2-negative and 57.5 [19.7] years for untested patients, Table 1). Medical facility characteristics and geographic regions by SARS-CoV-2 status are shown in Additional file 1: Table S1. During the pandemic, most patients who were tested for SARS-CoV-2 had specimens collected for microbial testing (89.5%), including 97.1% for patients who tested positive for SARS-CoV-2 and 88.8% for patients who tested negative. Among those not tested for SARS-CoV-2 during the pandemic, 40.0% of patients had specimens collected for microbiologic testing compared to 44.9% during the pre-pandemic period. The proportion of patients with GN/GP pathogens was 11.9% in SARS-CoV-2-positive patients, 11.0% in SARS-CoV-2-negative patients, and 8.3% in patients not tested.

**Table 1:** Patient demographics

	Pre-pandemic	SARS-CoV-2 +	SARS-CoV-2 -	SARS-CoV-2 not tested
Total admissions (N)	1,789,449	189,115	1,898,659	1,641,521
Male, n (%)	771,770 (43.1%)	95,001 (50.2%)*	832,409 (43.8%)*	705,186 (43.0%)*
Age (years), mean ± SD (median)	58.4 ± 19.7 (61)	61.5 ± 17.9 (63)*	58.5 ± 19.7 (62)*	57.5 ± 19.7 (60)*
Culture collected, n (%)	802,888 (44.9%)	183,658 (97.1%)*	1,685,161 (88. 8%)*	656,516 (40.0%)*
Hospital LOS (days), mean ± SD (median)	4.3 ± 5.8 (3)	7.9 ± 10.5 (5)*	4.8 ± 7.0 (3)*	3.9 ± 6.3 (2)*
ICU admissions, n (%)	213,616 (11.9%)	39,392 (20.8%)*	240,504 (12.7%)*	153,556 (9.4%)*
ICU LOS (days), mean ± SD (median)	3.5 ± 4.7 (2)	8.2 ± 9.7 (5)*	4.0 ± 5.6 (2)*	3.4 ± 4.8 (2)*

*ICU* intensive care unit, *LOS* length of stay, *SD* standard deviation

*P < 0.0008 compared with pre-pandemic period

**Table 2:** Trends by waves in GN/GP pathogens and antibiotic therapy for pre- and post-SARS-CoV-2 periods by SARS-CoV-2 testing status

Time period	SARS-CoV-2 +		SARS-CoV-2 −		SARS-CoV-2 not tested		Total admissions	
	Prescribed antibacterial therapy ≥ 24 h	Positive GN/GP culture during hospital stay	Prescribed antibacterial therapy ≥ 24 h	Positive GN/GP culture during hospital stay	Prescribed antibacterial therapy ≥ 24 h	Positive GN/GP culture during hospital stay	Prescribed antibacterial therapy ≥ 24 h	Positive GN/GP culture during hospital stay
Total: Jul 2019–Oct 2021	57.8%* (109,337/189,115)	11.9%* (22,558/ 189,115)	40.1% (761,398/ 1,898,659)	11.0%* (208,162/ 1,898,659)	32.8% (1,124,606/3,430,970)	8.3%* (285,279/ 3,430,970)	36.2% (1,995,381/ 5,518,744)	9.3% (515,999/ 5,518,744)
Baseline: Jul 2019–Feb 2020					35.0% (625,994/1,789,449)	9.3% (167,045/ 1,789,449)	35.0% (625,994/ 1,789,449)	9.3% (167,045/ 1,789,449)
Total: Mar 2020–Oct 2021	57.8% (109,337/189,115)	11.9% (22,558/ 189,115)	40.1% (761,398/ 1,898,659)	11.0% (208,162/ 1,898,659)	30.4% (498,612/ 1,641,521)	7.2% (118,234/ 1,641,521)	36.7% (1,369,387/ 2,359,908)	9.4% (348,954/ 3,729,295)
Mar 2020–May 2020	67.8% (10,643/15,702)	13.2% (2,067/ 15,702)	46.8% (54,681/ 116,755)	12.5% (14,541/ 116,755)	33.3% (132,120/396,348)	8.7% (34,422/396,348)	37.3% (197,444/528,805)	9.7% (51,030/ 528,805)
Jun 2020–Aug 2020	63.2% (16,862/26,699)	12.9% (3,443/ 26,699)	40.2% (130,724/ 324,814)	11.2% (36,312/ 324,814)	27.9% (73,288/262,947)	6.7% (17,740/262,947)	35.9% (220,874/614,460)	9.4% (57,495/ 614,460)
Sep 2020–Nov 2020	57.6% (15,025/26,081)	11.5% (2,995/ 26,081)	40.0% (135,412/ 338,745)	11.0% (37,135/ 338,745)	30.3% (70,141/231,131)	7.0% (16,225/231,131)	37.0% (220,578/595,957)	9.5% (56,355/ 595,957)
Dec 2020–Feb 2021	57.2% (33,076/57,819)	11.9% (6,873/ 57,819)	39.4% (126,140/ 319,865)	10.7% (34,346/ 319,865)	30.8% (57,306/186,155)	6.6% (12,194/186,155)	38.4% (216,522/563,839)	9.5% (53,413/ 563,839)
Mar 2021–May 2021	54.4% (10,686/19,645)	12.2% (2,392/ 19,645)	39.5% (139,349/ 353,050)	10.6% (37,445/ 353,050)	28.9% (66,789/231,491)	6.6% (15,191/231,491)	35.9% (216,824/604,186)	9.1% (55,028/ 604,186)
Jun 2021–Oct 2021	53.5% (23,085/43,169)	11.1% (4,788/ 43,169)	39.3% (175,092/ 445,430)	10.9% (48,383/ 445,430)	29.7% (98,968/333,449)	6.7% (22,462/333,449)	36.1% (297,145/822,048)	9.2% (75,633/ 822,048)

*GN* Gram-negative, *GP* Gram-positive

*P < 0.05 bivariate chi-squared correlation tests

### Antibiotic use and GN/GP positivity over time

During the pre-pandemic period (July 2019 through February 2020), 35% (625,994/1,789,449) admissions were prescribed antibacterial therapy for ≥ 24 h and 9.3% (167,045/1,789,449) of admissions were positive for a GN/GP pathogen (Table 2). In the pre-pandemic period, the median duration of antibacterial therapy was 3 days overall and 5 days in patients with a culture-positive GN/GP pathogen (Additional file 2: Table S2). Total admissions prescribed antibacterial therapy and those with a positive bacterial culture for GN/GP pathogens did not significantly change from the pre-pandemic through the pandemic periods over time (Table 3). The median duration of antibiotic therapy in the overall population and among patients with culture-positive GN/GP pathogens also did not change during the pandemic.

**Table 3:** Multivariate trends in percentage GN/GP-positive cultures and percentage of admissions prescribed antibiotics expressed as slope (95% CI)

Trend	Total		SARS-CoV-2 +		SARS-CoV-2-		Not tested	
	Slope (95% CI)	*P*	Slope (95% CI)	*P*	Slope (95% CI)	*P*	Slope (95% CI)	*P*
Slope in % positive GN/GP culture by wave	− 0.11 (− 0.26–0.05)	*P* = .269	− 0.38 (− 0.54–0.22)	*P* = 0.023	− 0.36 (− 0.81–0.03)	*P* = 0.048	− 0.37 (− 0.48–0.26)	*P* < .001
Slope in % prescribed antibiotic therapy by wave	− 0.08 (− 0.21–0.06)	*P* = 0.335	− 2.69 (− 3.06–2.31)	*P* < .001	− 1.69 (− 1.91–1.47)	*P* < .001	− 0.11 (− 0.28–0.06)	*P* = 0.203
Slope in % prescribed 3^rd^/4^th^ generation cepahlosporins by wave	− 0.02 (− 0.14–0.09)	*P* = 0.667	− 1.61 (− 1.98–1.25)	*P* < .001	− 1.18 (− 1.36–0.99)	*P* < .001	− 0.067 (− 0.183–0.048)	*P* = 0.252
Slope in % BLICs by wave	− 0.28 (− 0.35–0.21)	*P* < .001	− 0.37 (− 0.61–0.12)	*P* = 0.004	− 0.32 (− 0.45–0.19)	*P* < .001	− 0.16 (− 0.24–0.07)	*P* = 0.001
Slope in % glycopeptides by wave	− 0.22 (− 0.28–0.15)	*P* < .001	− 0.21 (− 0.40–0.02)	*P* = 0.047	− 0.34 (− 0.46–0.22)	*P* < .001	− 0.15 (− 0.22–0.07)	*P* < .001
Slope in % macrolides by wave	− 0.28 (− 0.40–0.16)	*P* < .001	− 3.78 (− 4.14–3.41)	*P* < .001	− 1.59 (− 1.76–1.43)	*P* < .001	0.08 (− 0.08–0.17)	*P* = 0.772

*BLIC* beta-lactam inhibitor combination, *CI* confidence interval, *ICU* intensive care unit, *LOS* length of stay, *GN* Gram-negative, *GP* Gram-positive

^*^All multivariate models control for % male, average age, LOS, % ICU, % ventilated, % ≥ 1 comorbid condition, teaching status, urban, bed size, and region

### Antibiotic use and GN/GP positivity by SARS-CoV-2 testing status

Rates of antibiotic use were substantially higher (*P* < 0.05) among SARS-CoV-2-positive patients (57.8%) compared to SARS-CoV-2-negative patients (40.1%) or patients not tested (32.8%). Positive GN/GP culture rates were 9.3% among all admissions and were highest (*P* < 0.05) in SARS-CoV-2-positive patients (11.9%), compared to 11.0% among SARS-CoV-2-negative patients and 8.3% among those not tested.

**Fig. 1:**
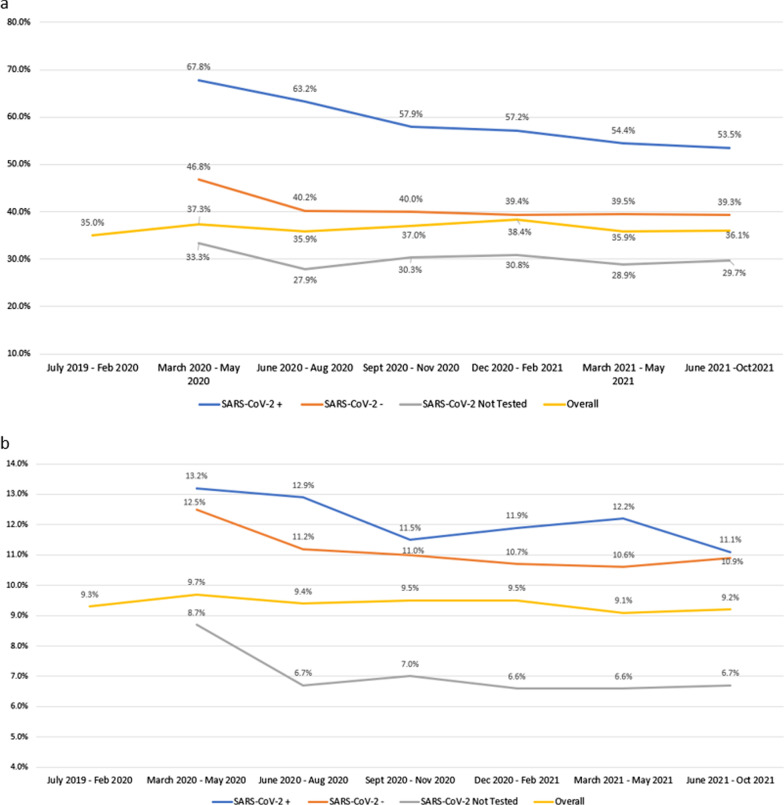
Trends in the percentage of admissions from July 2019–October 2021 by SARS-CoV-2 testing status for (**a**) admissions prescribed antibacterial therapy and (**b**) admissions with a GN/GP-positive pathogen

Overall rates of antibacterial use for ≥ 24 h were consistent across time periods in patients not tested. However, antibacterial use significantly decreased by an average of 2.69% each wave in SARS-CoV-2 positive patients (*P* < 0.001) and 1.69% in SARS-CoV-2 negative patients (*P* < 0.001) (Table 3, Fig. 1a). For example, among patients who tested positive for SARS-CoV-2, 67.8% received antibiotics between March and May 2020 and 53.5% received antibiotics between June and October 2021.

Overall admissions with a GN/GP-positive culture were consistent across the study period with pre-pandemic rates of 9.4% and 9.2% during the most recent study period. The rate of admissions with a GN/GP-positive culture was highest in SARS-CoV-2 positive patients, peaking at 13.2% during the March 2020 to May 2020 period and decreasing to 11.1% in the most recent period (Table 2; Fig. 1b). During the pandemic period, there was a significant 0.38% decrease in the percent of SARS-CoV-2-positive patients testing positive for GN/GP pathogens by wave (*P* = 0.023). A significant 0.36% decline in the percentage of SARS-CoV-2-negative patients prescribed antibiotics by wave was also observed, as was a decline of 0.37% (*P* < 0.001) in antibiotic prescriptions among patients not tested (Table 3).

The median duration of antibiotic use was consistent across study periods but varied by SARS-CoV-2 testing status and by GN/GP-culture status. Among SARS-CoV-2-positive patients, the median duration of antibiotic use was 5 days throughout the study period. The median duration of antibiotic therapy was 4 days in SARS-CoV-2-negative patients and 3 days in patients who were not tested. The duration of antibiotic therapy was greater among patients with positive GN/GP cultures across all SARS-CoV-2 testing groups. For example, among patients with GN/GP culture-positive results, median duration of antibiotic therapy was steady at 5.0 days.

The use of different classes of antibiotic therapy was highest in all groups during the early pandemic period (between March and May 2020) and then declined over time, with the largest declines in SARS-CoV-2 positive patients (Table 4). This was most notable for macrolides and 3^rd^/4^th^ generation cephalosporins, which both showed a significant (*P* < 0.001) average decline of 3.78% and 1.61%, respectively, for SARS-CoV-2-positive patients. However, no declines were observed among patients not tested during the pandemic period (Table 3).

**Table 4:** Trends in percentage of admissions prescribed antibiotics (95% CI)

Time period	Total		SARS-CoV-2 +		SARS-CoV-2-		Not tested	
	Adjusted % positive GN/GP culture	% Prescribed Abx therapy ≥ 24 h	Adjusted % positive GN/GP culture	% Prescribed Abx therapy ≥ 24 h	Adjusted % positive GN/GP culture	% Prescribed Abx therapy ≥ 24 h	Adjusted % positive GN/GP culture	% Prescribed Abx therapy ≥ 24 h
Pre-pandemic: July 2019–Feb 2020	9.49 (8.86–10.13)	36.66 (35.46–37.87)	NA	NA	NA	NA	NA	NA
March 2020–May 2020	9.61 (8.98–10.24)	37.76 (36.97–38.55)	13.34 (12.45–14.23)	68.02 (66.57–69.46)	12.23 (11.32–13.13)	46.99 (45.49–48.49)	8.51 (7.88–9.13)	32.48 (31.15–22.81)
June 2020–August 2020	9.38 (8.75–10.01)	35.50 (34.31–36.69)	12.89 (11.77–14.00)	62.82 (60.55–65.08)	11.08 (10.17–11.99)	40.30 (38.87–41.73)	6.81 (6.17–7.44)	29.44 (28.11–20.78)
Sept 2020–Nov 2020	9.57 (8.95–10.20)	36.89 (35.70–38.08)	11.60 (10.52–12.69)	57.49 (54.27–58.72)	10.99 (10.03–11.94)	40.00 (38.56–41.44)	6.92 (6.29–7.55)	30.90 (29.57–32.24)
Dec 2020–Feb 2021	9.69 (9.06–10.33)	38.43 (37.24–39.62)	11.69 (10.75–12.63)	57.31 (55.11–59.51)	10.88 (9.95–11.80)	39.57 (38.12–41.01)	6.71 (6.08–7.34)	31.67 (30.33–33.01)
March 2021–May 2021	9.17 (8.53–9.81)	36.01 (34.81–37.21)	11.72 (10.76–12.68)	54.10 (51.84–56.36)	10.38 (9.47–11.30)	39.48 (38.02–40.95)	6.50 (5.87–7.14)	30.13 (28.78–31.48)
June 2021–October 2021	9.35 (8.76–9.94)	36.18 (35.05–37.32)	10.99 (9.73–12.25)	53.59 (51.50–55.67)	10.93 (10.07–11.80)	39.38 (38.01–40.74)	6.61 (6.02–7.20)	31.01 (29.73–32.29)

*Abx* antibiotic, *GN* Gram-negative, *GP* Gram-positive

## Discussion

This is among the first studies to evaluate national changes in inpatient GN/GP positivity and antibiotic use from July 2019, 8 months prior to the onset of the COVID-19 pandemic, through multiple waves of the pandemic. Our data demonstrate that while inpatient antibiotic use temporarily increased early in the pandemic, overall inpatient antibiotic use or GN/GP-positivity rates did not change from July 2019 through October 2021. Total adjusted inpatient GN/GP positivity rates were 9.49% during the pre-pandemic period and 9.35% between June and October 2021, while adjusted rates of antibiotic use during the same time periods were 36.7% and 36.2%, respectively.

Our data suggest that the overall use of antibiotics among inpatients appear to be lower than the results of Centers for Disease Control and Preventions’ (CDC) Emerging Infections Program (EIP) 2015 prevalence surveys, which found that approximately 49.5% of inpatients received antimicrobials [15]. Differences in results may be due to differences in data collection methods, the types of antimicrobials evaluated (antibacterial and selected antimycobacterial and antiviral medications in the CDC EIP study vs antibacterials only in our study), how use was defined, or to recent inpatient antimicrobial stewardship efforts. A more recent study of antibiotic use and presumptive pathogens in Veterans Affairs acute care facilities reported antibiotic use in 41.9% of all hospital stays between October 2017 and September 2018, with 10.2 to 31.4% of antibiotic days of therapy linked to a potential bacterial pathogen [16].

Our results confirmed the findings of other studies that demonstrated an initial increase in antibiotic use during the early pandemic period, particularly among SARS-2-CoV-positive patients [3, 4, 17], followed by a subsequent decrease in later pandemic periods [3]. Rates of GN/GP positivity in SARS-2-CoV-positive patients were 13.3% between March 2020 and May 2020, which decreased to 11.0% between June 2021 and October 2021, while rates of antibacterial antibiotics prescribed during hospitalization decreased from 68.0 to 53.6% over the same period. Similar decreases were observed in SARS-CoV-2-negative patients.

Although increased antibiotic use in the early wave of the pandemic (March 2020 to May 2020) in our study was driven by an increase in use of all 4 evaluated classes of antibiotic (3rd and 4th generation cephalosporins, beta-lactamase inhibitors, glycopeptides, and macrolides), more than half (52.1%) of SARS-CoV-2 + admissions received 3rd or 4th generation cephalosporins. However, the use of all classes of antibiotics decreased in SARS-2-CoV-positive patients in later stages of the pandemic. Similar trends were noted in SARS-CoV-2-negative patients.

A rapid review and meta-analyses of 154 studies evaluating antibiotic prescribing in patients with COVID-19 (N = 35,263) from the onset of the pandemic through June 2020 estimated that overall antibiotic use was 74.6% [3]. However, as noted in our study, a trend towards reduced antibiotic prescribing as the pandemic progressed was reported, from 85.8% in studies conducted before January 2020 to 62.6% for studies ending in April 2020 [3].

In a study of 213,338 inpatients diagnosed with COVID-19 in 716 hospitals between March and October 2020 in the Premier Healthcare Database, 77.3% were billed for antibiotic therapy for at least 1 day [18]. However, overall hospital-wide antibiotic use was significantly lower between March and October 2020 compared with March and October 2019, particularly among hospitals with the lowest burden of COVID-19. Hospitals with a higher burden of COVID-19 reported increased use of ceftriaxone, cefepime, and azithromycin, as we observed in SARS-2-CoV-positive patients in our study. The use of vancomycin, piperacillin-tazobactam, and levofloxacin was consistently lower in 2020 vs 2019, regardless of COVID-19 burden, suggesting a possible decline in empiric prescribing on admission and throughout hospitalization.

Our study also confirms the results of numerous previous studies that describe the mismatch between the high levels of antimicrobial use and low levels of confirmed bacterial infections in patients SARS-CoV-2-positive patients [1–4, 9, 18–21]. Our results also may provide insights on the frequency of antibiotic use and GN/GP-positive infections among SARS-CoV-2-negative patients and patients not tested for SARS-CoV-2, which appear to be similar to those observed prior to the pandemic and may help inform future inpatient antimicrobial and diagnostic stewardship efforts.

While our study was not designed to evaluate the appropriateness of antibiotic therapy, we suspect that the increased use of antibiotic therapy among SARS-2-CoV-positive patients early in the pandemic was driven by previous experience with coinfections during influenza outbreaks and potentially by an uncertainty about how to manage severe COVID-19 [1, 4, 19]. However, rates of antibiotic prescription in the absence of a documented infection remained high even during the summer and early fall of 2021, when clinicians were more familiar with how to manage COVID-19 and immunosuppressive strategies for managing COVID-19 were more widely available.

High rates of antibiotic use, particularly in the absence of a documented bacterial infection, can increase rates of antimicrobial resistant (AMR) infections. In a previous analysis of the same dataset, we demonstrated that hospitalized patients tested for SARS-CoV-2 infection exhibited a significantly higher rate of antimicrobial resistant (AMR) infections compared to pre-pandemic rates, although overall AMR rates in hospitalized adults did not substantially increase from pre-pandemic levels [11]. Factors associated with increased AMR included increases in overall antibiotic use, rates of positive cultures, duration of antibiotic therapy, and use of inadequate empiric therapy. We also demonstrated that use of inadequate empiric therapy among patients hospitalized during the pandemic was associated with a 21% increase in mortality and longer lengths of stay compared with patients who received adequate empiric therapy, both in the overall population tested for SARS-CoV-2 and among patients who tested positive and negative for SARS-CoV-2 [13].

Given that 41.1% of SARS-CoV-2-positive patients without a positive culture in the analysis received antibacterial therapy for ≥ 72 h, timely review of antimicrobial prescribing as part of antimicrobial stewardship programs that is coupled with enhanced diagnostic stewardship can reduce the selective pressures for antimicrobial resistance. Further study of recent trends and outcomes in antibiotic use among inpatients is warranted given changes in the severity and transmissibility of SARS-CoV-2 infection since emergence of the Omicron variant, the widespread adoption of SARS-CoV-2 vaccination and boosters, and a greater number of therapeutic options for SARS-CoV-2 infections.

This study has several advantages. First, the study includes data from later stages of the pandemic, data derived from a period in which vaccination was widely accessible and when clinicians became increasingly knowledgeable about managing COVID-19. The study also includes data from the period in which clinically relevant variants became increasingly dominant in the United States, including Delta, and patients from a variety of hospitals in geographically diverse regions, which may provide a more complete view of and clinical practice nationwide.

Our study also has several limitations. First, SARS-CoV-2 and pathogen status were based on reports from individual facilities, there was no standard testing method, and no central laboratory was used. No case definition for COVID-19 disease was applied, so it is possible that some SARS-CoV-2-positive patients were asymptomatic and admitted for other reasons. While our algorithm was designed to exclude patients admitted with colonizing microbes [14], some patients without clinically significant infections may have been included. Finally, because influenza was uncommon during our observation period, our pre-pandemic data may not be representative of other time periods.

## Conclusion

Although antibiotics may have been overused among hospitalized SARS-CoV-2-positive patients during the early wave of the pandemic, decreases in the frequency of antibiotic prescriptions were observed in later waves. Overall antimicrobial use and incidence of culture-positive GN/GP did not change from the pre-pandemic through the pandemic period evaluated. These results, combined with results from previous analyses, may inform opportunities for stewardship programs to refine antibiotic prescribing in the current pandemic and to help inform antibiotic use in future viral outbreaks.

## Supplementary Information

**Additional file 1: Table S1.** Hospital demographics.

**Additional file 2: Table S2.** Duration of antibacterial therapy overall and in admissions with a GN/GP-positive pathogen from July 2019–October 2021 by SARS-CoV-2 testing status.

## Data Availability

The datasets used and/or analyzed during the current study are included in the tables in the main manuscript and additional information.
