# Supplementary material for: A multicenter evaluation of antibacterial use in hospitalized patients through the SARS-Cov-2 pandemic waves
Source: BMC Infect Dis. 2023 Feb 24;23:117. doi: 10.1186/s12879-023-08042-0 (PMC9951830; doi:10.1186/s12879-023-08042-0)
Supplement: Supplementary file 1 — Additional file 1: Table S1. Hospital demographics. [file 12879_2023_8042_MOESM1_ESM.docx]

**Additional file 1: Table S1.** Hospital demographics

| **Hospital Demographics** | **N (%)** |
| --- | --- |
| **Total** | **271 (100%)** |
| **Bed size** |  |
| < 100 | 96 (35.42%) |
| 100-300 | 108 (39.85%) |
| > 300 | 67 (24.72%) |
| Urban | 177 (65.31%) |
| Rural | 94 (34.69%) |
| Teaching | 90 (33.2%) |
| Non-teaching | 181 (66.79%) |
| **Census Region** |  |
| East North Central | 42 (15.50%) |
| East South Central | 36 (13.28%) |
| Middle Atlantic | 38 (14.02%) |
| Mountain | 11 (4.06%) |
| New England | 5 (1.85%) |
| Pacific | 27 (9.96%) |
| South Atlantic | 41 (15.13%) |
| West North Central | 16 (5.90%) |
| West South Central | 55 (20.30%) |
