# Supplementary material for: A multicenter evaluation of antibacterial use in hospitalized patients through the SARS-Cov-2 pandemic waves
Source: BMC Infect Dis. 2023 Feb 24;23:117. doi: 10.1186/s12879-023-08042-0 (PMC9951830; doi:10.1186/s12879-023-08042-0)
Supplement: Supplementary file 2 — Additional file 2: Table S2. Duration of antibacterial therapy overall and in admissions with a GN/GP-positive pathogen from July 2019–October 2021 by SARS-CoV-2 testing status. [file 12879_2023_8042_MOESM2_ESM.docx]

**Additional file 2: Table S2.** Duration of antibacterial therapy overall and in admissions with a GN/GP-positive pathogen from July 2019 – October 2021 by SARS-CoV-2 testing status

| Time Period | SARS-CoV-2 + | | SARS-CoV-2 - | | SARS-CoV-2 Not Tested | | Total Admissions | |
| --- | --- | --- | --- | --- | --- | --- | --- | --- |
|  | Prescribed Antibacterial Therapy ≥ 24 hours | Positive GN/GP Culture During Hospital Stay | Prescribed Antibacterial Therapy ≥ 24 hours | Positive GN/GP Culture During Hospital Stay | Prescribed Antibacterial Therapy ≥ 24 hours | Positive GN/GP Culture During Hospital Stay | Prescribed Antibacterial Therapy ≥ 24 hours | Positive GN/GP Culture During Hospital Stay |
| Total: July 2019 - October 2021: Avg Abx Duration (Days)*: avg ± sdev (med; n) | 6.4 ± 6.0 (5; 109,337)* | 10.0 ± 9.0 (7; 20,267)* | 5.0 ± 5.1 (4; 761,398)* | 7.2 ± 7.1 (5; 183,259)* | 4.5 ± 4.6 (3; 1,124,606)* | 6.4 ± 6.4 (5; 241,656)* | 4.8 ± 4.9  (3; 1,995,381) | 6.9 ± 6.9 (5; 445,182) |
| Baseline: July 2019 - February 2020: Avg Abx Duration (Days)*: avg ± sdev (med; n) |  | | | | 4.7 ± 4.7 (3; 625,994) | 6.6 ± 6.6 (5; 143,790) | 4.7 ± 4.7 (3) | 6.6 ± 6.6 (5; 143,790) |
| Total: March 2020 - October 2021: Avg Abx Duration (Days)*: avg ± sdev (med, n) | 6.4 ± 6.0 (5; 109,337) | 10.0 ± 9.0 (7; 20,267) | 5.0 ± 5.1 (4; 761,398) | 7.2 ± 7.1 (5; 183,259) | 4.3 ± 4.4 (3; 498,612) | 6.1 ± 6.2 (4; 97,866) | 4.9 ± 5.0 (3; 1,369,387)) | 7.0 ± 7.0 (5; 348,954) |
| March 2020 - May 2020: Avg Abx Duration (Days)*: avg ± sdev (med, n) | 6.1 ± 5.4 (5; 10,643) | 9.5 ± 8.2 (7; 1,866) | 5.1 ± 5.0 (4; 54,681) | 7.1 ± 7.1 (5; 13,108) | 4.9 ± 5.3 (3; 132,120) | 7.1 ± 7.5 (5; 29,424) | 5.0 ± 5.3 (3; 197,444) | 7.2 ± 7.5 (5; 44,398) |
| June 2020 - August 2020: Avg Abx Duration (Days)*: avg ± sdev (med, n) | 6.6 ± 6.4 (5; 16,862) | 11.2 ± 9.9 (8; 3,185) | 5.0 ± 5.1 (4; 130,724) | 7.2 ± 6.9 (5; 32,313) | 4.0 ± 4.0 (3; 73,288) | 5.3 ± 5.3 (4; 14,276) | 4.8 ± 4.9 (3; 220,874)) | 6.9 ± 6.9 (5; 49,774) |
| September 2020 - November 2020: Avg Abx Duration (Days)*: avg ± sdev (med, n) | 6.3 ± 5.9 (5; 15,025) | 9.9 ± 8.9 (7; 2,712) | 5.0 ± 5.0 (3; 135,412) | 7.1 ± 6.7 (5; 32,822) | 4.1 ± 4.0 (3; 70,141) | 5.5 ± 5.1 (4; 13,339) | 4.8 ± 4.8 (3; 220,578) | 6.8 ± 6.6 (5; 48,873) |
| December 2020 - February 2021: Avg Abx Duration (Days)*: avg ± sdev (med, n) | 6.3 ± 5.7 (5; 33,076) | 9.4 ± 8.1 (7; 6,128) | 5.1 ± 5.2 (4; 126,140) | 7.2 ± 7.1 (5; 30,086) | 4.4 ± 4.3 (3; 57,306) | 5.9 ± 5.5 (4; 10,030) | 5.1 ± 5.2 (4; 216,522) | 7.2 ± 7.0 (5; 46,244) |
| March 2021 - May 2021: Avg Abx Duration (Days)*: avg ± sdev (med, n) | 6.8 ± 7.0 (5; 10,686) | 10.9 ± 10.4 (8; 2,138) | 5.0 ± 5.3 (3; 139,349) | 7.3 ± 7.4 (5; 33,037) | 4.2 ± 4.2 (3; 66,789) | 5.8 ± 5.4 (4; 12,452) | 4.9 ± 5.1 (3; 216,824) | 7.0 ± 7.2 (5; 47,627) |
| June 2021 - October 2021: Avg Abx Duration (Days)*: avg ± sdev (med, n) | 6.4 ± 6.0 (5; 23,085) | 10.1 ± 9.1 (7; 4,238) | 5.0 ± 5.2 (3; 175,092) | 7.1 ± 7.3 (5; 41,893) | 4.1 ± 4.1 (3; 98,968) | 5.7 ± 5.6 (4; 18,345) | 4.8 ± 5.0 (3; 297,145) | 6.8 ± 7.1 (5; 64,476) |

**P*<0.05 ANOVA analysis of variance test compared to total admissions.

Abbreviations: Abx, antibiotics; ANOVA, Analysis of Variance; GN, Gram-negative; GP, Gram-positive.
